# Strain Tunable Bandgap and High Carrier Mobility in SiAs and SiAs_2_ Monolayers from First-Principles Studies

**DOI:** 10.1186/s11671-018-2809-6

**Published:** 2018-12-12

**Authors:** Shouyan Bai, Chun-Yao Niu, Weiyang Yu, Zhili Zhu, Xiaolin Cai, Yu Jia

**Affiliations:** 1 International Laboratory for Quantum Functional Materials of Henan, Zhengzhou University, Zhengzhou, 450001 People’s Republic of China; 2 School of Physics and Electronic Information Engineering, Henan Polytechnic University, Jiaozuo, 454000 People’s Republic of China; 3 Key Laboratory for Special Functional Materials of Ministry of Education, Henan University, Kaifeng, 475001 People’s Republic of China

**Keywords:** SiAs, Two-dimensional semiconductors, Higher carrier mobility, First-principles

## Abstract

Searching for new stable free-standing atomically thin two-dimensional (2D) materials is of great interest in the fundamental and practical aspects of contemporary material sciences. Recently, the synthesis of layered SiAs single crystals has been realized, which indicates that their few layer structure can be mechanically exfoliated. Performing a first-principles density functional theory calculations, we proposed two dynamically and thermodynamically stable semiconducting SiAs and SiAs_2_ monolayers. Band structure calculation reveals that both of them exhibit indirect band gaps and an indirect to direct band even to metal transition are found by application of strain. Moreover, we find that SiAs and SiAs_2_ monolayers possess much higher carrier mobility than MoS_2_ and display anisotropic transportation like the black phosphorene, rendering them potential application in optoelectronics. Our works pave a new route at nanoscale for novel functionalities of optical devices.

**Electronic supplementary material:**

The online version of this article (10.1186/s11671-018-2809-6) contains supplementary material, which is available to authorized users.

## Background

Atomically thin two-dimensional (2D) crystals have become one of the most rapidly burgeoning field of contemporary material science. The versatile electronic properties, excellent electron mobility, and promising applications in nanoelectronics and optoelectronics are driving a large percentage of condensed-matter physicists to hunt for new 2D materials. Following graphene [1–4], a huge number of other 2D materials have been synthesized such as silicene [5–7], boron-nitride nanosheets [8, 9], transition-metal dichalcogenides (TMDs) [10, 11], black phosphorus [12, 13], borophene [14–16], arsenene [17, 18], tellurene [19], and their isoelectronic compounds [20–23]. The list of 2D materials is fast expanding, and more than thousands kinds of such materials are now known, encompassing the full spectrum of electronic and other properties. And their novel properties, different from or even better than those of their bulk counterparts, are theoretically predicted and experimentally confirmed firmly.

Although extensive and substantial efforts were invested in finding diverse 2D materials including some that already possess bandgaps or other desirable properties, consensus has not been reached. Graphene with marvelous carrier mobility, high mechanical stability, and massless dirac electrons has attracted much attention to date, but the lacking of an intrinsic band gap hinder its application in modern electronic devices industry. Although large efforts have been made, opening up a sizeable band gap without side-effect has not been reached [24, 25]. TMDs with high performance in optoelectronic devices indeed have intrinsic band gap, but exhibit poor in carrier mobility [26–28]. Black and blue phosphorus with a strain sensitive tunable band gap and anisotropic high carrier mobility can not keep stable in air [13, 29]. Recently, the synthesis of layered SiAs and SiAs_2_ single crystals has been realized [30–32], which indicates that few layer structure can be obtained by mechanically exfoliated.

In the present work, based on a first-principles density functional theory calculations (DFT), we proposed two dynamically and thermodynamically stable semiconducting monolayers SiAs and SiAs_2_. They both possess indirect band gaps (2.39 eV and 2.13 eV respectively). Application of isotropic strain along two in-plane directions practically transforms the SiAs (SiAs_2_) monolayer into a direct-gap 1.75 eV (1.60 eV) material. Moreover, we find that SiAs and SiAs_2_ monolayers possess much higher carrier mobility than MoS_2_ and display anisotropic transportation like the black phosphorene, rendering them potential application in optoelectronics. Our works paves a new route at nanoscale for novel functionalities of optical devices.

## Computational Methods

The DFT calculations are performed using Vienna ab initio simulation package (VASP) code [33]. We used the Perdew-Burke-Ernzerhof (PBE) [34] exchange-correlation functional under the generalized gradient approximation(GGA). The projector augmented wave (PAW) method [35] was employed to describe the electron-ion interaction. A vacuum of 20 Å perpendicular to the sheets (along the c axis) was applied to avoid the interaction between layers. A kinetic energy cut-off of 500 eV is used for the plane wave basis set. The Brillouin-zone sampling is carried out with a 15 × 5 × 1 Monkhorst-Pack [36] grid for 2D sheets. Convergence criteria employed for both the electronic self-consistent relaxation and ionic relaxation are set to be 10^−4^ and 0.01 eV/Å for energy and force, respectively. The phonon calculations are carried out using the supercell method through the PHONOPY code [37, 38], and the real-space force constants of supercells are calculated in the density-functional perturbation theory (DFPT) as implemented in VASP. Moreover, a more strict energy (10^−8^ eV/atom) and force convergence criterion (10^−4^ eV/Å) are used during the vibrational spectra calculations. In the molecular dynamics(MD) calculations, (3×3×1) supercells are employed and the temperature is kept at 300 K for 6 ps with a time step of 2 fs in the moles-volume-temperature (NVT) ensemble. The raman spectra were calculated at the PBE level of theory using the CASTEP code [39–41].

## Results and Discussions

**Fig. 1:**
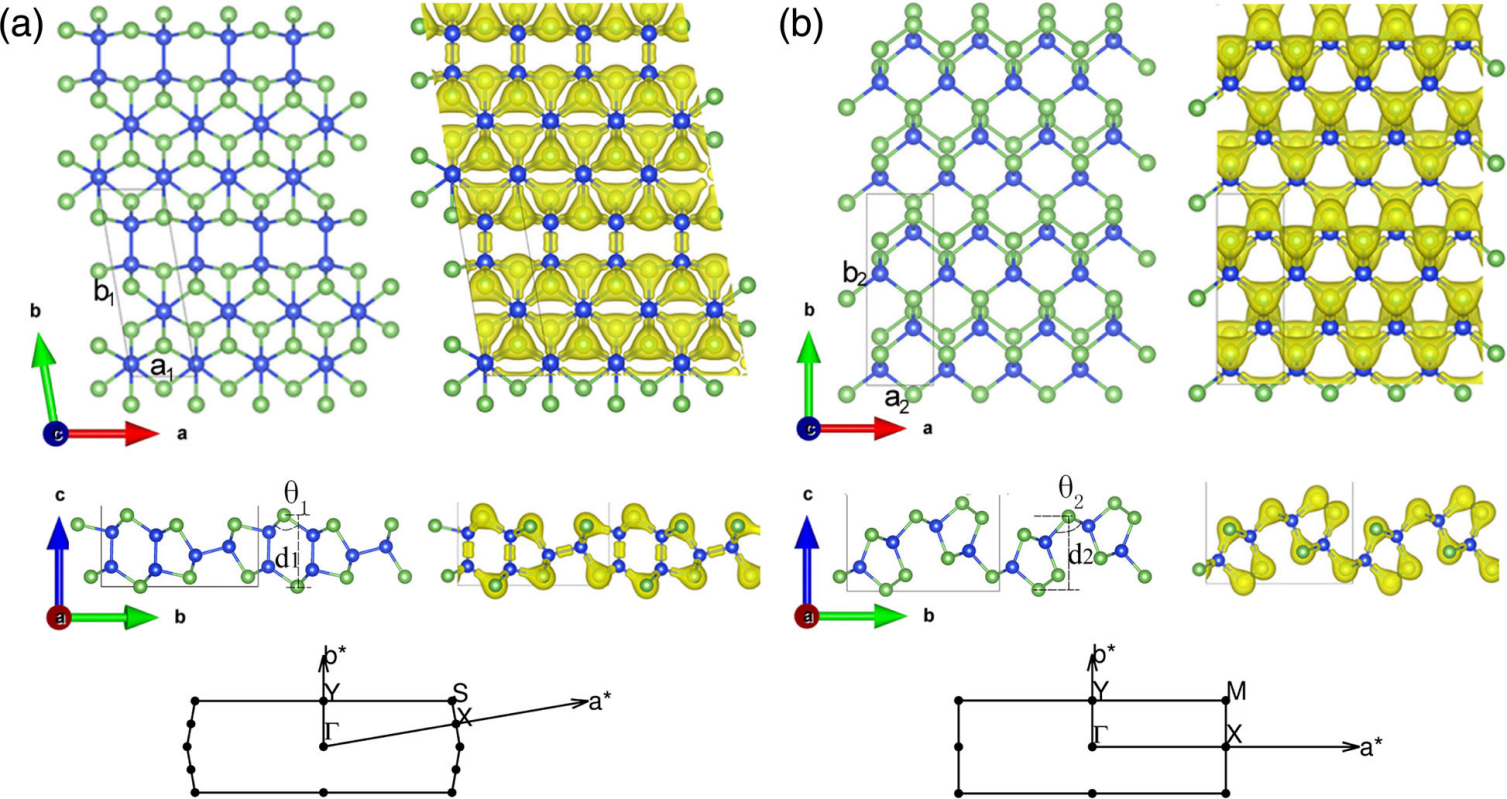
Geometrical structure and electron density distribution of monolayers SiAs and SiAs_2_. (Color online) Top and side views of monolayers **a** SiAs and **b** SiAs_2_ geometrical structure and electron density distribution and the associated Brillouin zone. The blue and green ball indicate the Si and As atom, respectively

The geometrical structures and electron density distribution of relaxed free-standing 2D SiAs and SiAs_2_ are presented in Fig. 1a, b, respectively, and their bulk structures are shown in Additional file 1: Figure S1 of the supplementary material. As shown in Additional file 1: Figure S1a and b, the bulk SiAs(SiAs_2_) possesses C2/m(Pbam) symmetry and consists of stacked Si-As layers weakly bound by van der Waals forces with a distance of 3.06 Å (1.66 Å). The unit cell of monolayer SiAs is rhombic and its optimized crystal parameters are *a*_1_ = 3.69Å and *b*_1_ = 10.83Å with *φ*=99.81°. SiAs contains 6 Si atoms and 6 As atoms. Each Si atom has four nearest neighboring atoms (3 As and 1 Si) while each As atom forms only three covalent bonds with neighboring Si atoms. It exists two kinds of bonds, namely, Si–Si and Si–As bonds. And the Si–Si bond length is about 2.35 Å and that of Si–As is in the range of 2.39 Å and 2.43 Å, and the buckled height is *d*_1_ = 4.86 Å. At the side view of monolayer SiAs, a eyeglass-stringed like structure is formed with double and single layers alternately bulked. Another monolayer structure of silicon and arsenic compound is SiAs_2_. Its prime cell contains 4 Si atoms and 8 As atoms, with a rectangular structure and the optimized crystal parameters are *a*_2_ = 3.68Å and *b*_2_ = 10.57 Å. Each As atoms has three nearest neighboring Si atoms or forms one covalent bond with neighboring Si atoms and two covalent bonds with themselves, while each Si atoms has only four nearest neighboring As atoms. Unlike the former, SiAs_2_ owns weaker As–As bond (2.50 Å) instead of Si–Si bond. And its Si–As bonds range from 2.41 Å to 2.45 Å, and the buckled height is *d*_2_ = 5.09 Å. From the electron density distribution, the As atoms attract electron from Si Atoms for their large electronegativity and have a larger electron density. In order to assist future experimental characterization, we further calculated and checked the Raman spectra of bulk and monolayer SiAs and SiAs_2_. Clear shifts between the monolayer and the full crystals have been seen in Additional file 1: Figure S2 of the supplementary material, whose origins have been identified as the influence of layers van der Waals interaction [42].

To learn the stability of SiAs (SiAs_2_), we first calculated the cohesive energy, defined as *E*_coh_ = (*nE*_Si_ + *mE*_As_−*E*_Mono_)/(*n* + *m*), where *E*_Si_,*E*_As_, and *E*_Mono_ are the total energies of a single Si atom, a single As atom, and one formula unit of monolayer SiAs (SiAs_2_), respectively, and n(m) is the number of As(Si) atom in the formula unit. Our calculations show that the SiAs monolayer has a cohesive energy of 5.13 eV/atom, which is a bit larger than that of SiAs_2_ monolayer 4.98 eV/atom. For comparison, at the same theoretical level, the cohesive energies of arsenene and silicene are 2.99 and 3.71 eV/atom, respectively [18, 43]. The high cohesive energies of SiAs and SiAs_2_ reveal that both of them are bonded strongly with high stability.

**Fig. 2:**
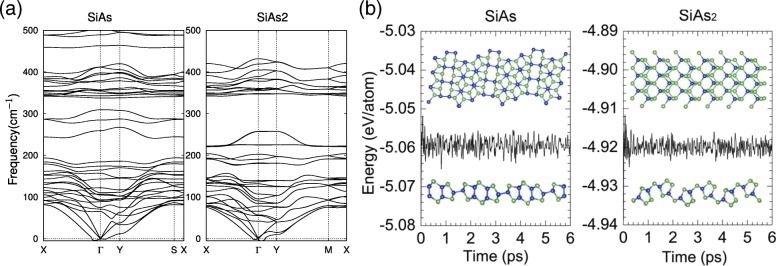
Phonon dispersion curves and MD simulations of monolayers SiAs and SiAs_2_. **a** The phonon dispersion curves for monolayer SiAs and SiAs_2_. **b** Relationships of total energy and time during room-temperature MD simulations of SiAs and SiAs_2_. Selected snapshots of the monolayer structures at the end of 6 ps are also provided

To further confirm the structural stabilities of monolayer SiAs and SiAs_2_, we also have performed vibrational phonon spectra calculations. As shown in Fig. 2a, positive frequencies account for a majority of modes except the transverse acoustic mode near the *Γ* point, which is due to the softening of phonons and has been reported in other similar systems [44, 45], indicating that the structures are both dynamically stable. Then, we carried out 6 ps first-principles MD simulations at room temperature (*T*=300*K*), as presented in Fig. 2b. The slight energy fluctuation and well kept sturctures suggest that they are thermally stable at room temperature. Our results imply that the monolayers SiAs and SiAs_2_ could be realized experimental at room temperature.

**Fig. 3:**
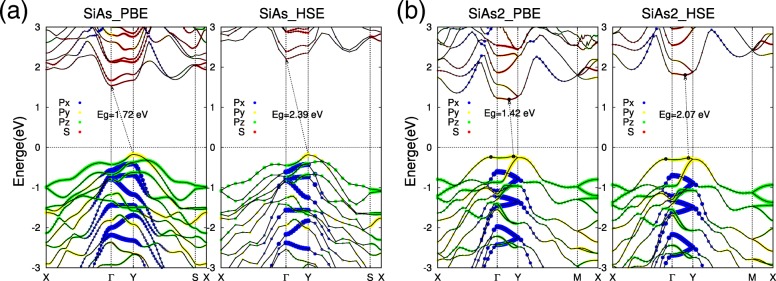
Band structures of monolayer SiAs and SiAs_2_ calculated by PBE and HSE06. The electronic orbital decomposition of band structures of monolayers SiAs and SiAs_2_ are represented as **a** and **b**, respectively. The red dots denote *s* orbital, while blue, yellow, and green are *p*_*x*_,*p*_*y*_, and *p*_*z*_, respectively. The Fermi level is set as zero and indicated with a dot line

With the optimized structures of monolayer SiAs and SiAs_2_, now we pay attention to their electronic properties. The calculated orbital decomposition band structures of SiAs and SiAs_2_ monolayers are shown in Fig. 3. Our calculations clearly show that SiAs and SiAs_2_ monolayers are both indirect semiconductors with wide band gaps. For monolayer SiAs, the valence band maximum (VBM) is located at the *Y* point, while the conduction band minimum (CBM) is at the *Γ* (Fig. 3a). The indirect band gap of monolayer SiAs is *E*_*g*_ = 1.72 eV within the PBE scheme. One can also see that the VBM state at *Y* point comprises the *p*_*y*_ orbital, while the CBM of *Γ* point comprises mainly the s orbital, which means that the external deformation will have different effects on the two states and may lead to indirect–direct transition, as revealed in the following. Unlike SiAs, the monolayer SiAs_2_ is a nearly direct semiconductor with VBM located at side of the *Y* point and CBM is of a little displacement from it (Fig. 3b). The SiAs_2_ monolayer indirect band gap is *E*_*g*_ = 1.42 eV within the PBE scheme. And the VBM and CBM of SiAs_2_ monolayer are comprised of the *p*_*y*_ orbital and s orbital, respectively. In order to get more accurate band gap value, we also performed the hybrid functional calculations (HSE06)[46, 47] for SiAs and SiAs_2_ monolayers. From the calculated band structures (the right part of Fig. 3a, b), the sharps of band states from PBE and HSE are basically the same, and the indirect band gap is still predicted within the hybrid functional calculations, but the gap value is increased to 2.39 eV and 2.07 eV for SiAs and SiAs_2_, respectively.

The carrier mobilities, which is a key factor of the potential applications in modern electronic devices for the newly discovered 2D materials, is as important as the bandgap and location of CBM and VBM. To gain more details on the electronic structure properties of SiAs and SiAs_2_ monolayers, we then calculated their acoustic phonon-limited carrier mobilities (including electron and hole in both x and y directions) on the basis of deformation potential (DP) theory [48] at the room temperature (*T*=300 *K*). At the low-energy regime (300 *K*), the electron-acoustic-phonon scattering dominates the carrier transport, which makes the acoustic phonon-limited an effective way to predict the carrier mobilities of many 2D structures, such as the MoS_2_ monolayer [49], tellurene [19], phosphene [50], and few-layer MoO_3_ [51]. The calculated effective masses *m*^∗^ and carrier mobilities *μ* of SiAs and SiAs_2_ monolayers show that both of them are high-mobility and transport anisotropy (see Additional file 1: Table S1 and the Figures S3 and S4) like the black phosphorene [50]. To estimate the carrier mobility of SiAs and SiAs_2_, we firstly performed a fit of their bands using the nearly free electron model to get the effective carrier masses. For SiAs, we define *x* and *y* as the direction perpendicular to the lattice vectors *b* and *a*, respectively. The $m_{e}^{*}$ and $m_{h}^{*}$ along the x direction are about 0.15 *m*_0_ and 0.86 *m*_0_, respectively, and along the y direction are 0.80 *m*_0_ and 0.22 *m*_0_ (*m*_0_ is the free-electron mass), respectively. For SiAs_2_, the direction of lattice vector *a* is defined as *x*, while that of *b* is *y*. The $m_{e}^{*}$ and $m_{h}^{*}$ along the x direction are about 0.14 *m*_0_ and 0.65 *m*_0_, respectively, and along the y direction are 2.05 *m*_0_ and 1.82 *m*_0_, respectively. We further studied the elastic constants (C) and the deformation potentials (E1) (see Additional file 1: Figure S2 and S3). Based on the above obtained *m*^∗^, C and E1 values, we estimated the carrier mobility as listed in Table 1. The electron mobilities for SiAs(SiAs_2_) along *x* and *y* directions are 0.66(0.26) and 0.54(0.11) × 10^3^ · *cm*^2^*V*^−1^*S*^−1^, while the hole mobilities along *x* and *y* directions are 3.90(0.13) and 0.30(0.65) × 10^3^ · *cm*^2^*V*^−1^*S*^−1^, respectively, both of which are much higher than those of MoS_2_ [49].

**Table 1:** Effective masses *m*^∗^ and carrier mobilities *μ* of SiAs and SiAs_2_, obtained using the PBE calculation at T = 300K

	*m*^∗^ (*m*_*e*_)		*μ*(10^3^ cm^2^V^−1^*s*^−1^)	
	Electron	Hole	Electron	Hole
SiAs	0.15 (*x*)	0.86 (*x*)	0.66 (*x*)	3.90 (*x*)
	0.80 (*y*)	0.22 (*y*)	0.54 (*y*)	0.30 (*y*)
SiAs_2_	0.14 (*x*)	0.65 (*x*)	0.26 (*x*)	0.13 (*x*)
	2.05 (*y*)	1.82 (*y*)	0.11 (*y*)	0.65 (*y*)

**Fig. 4:**
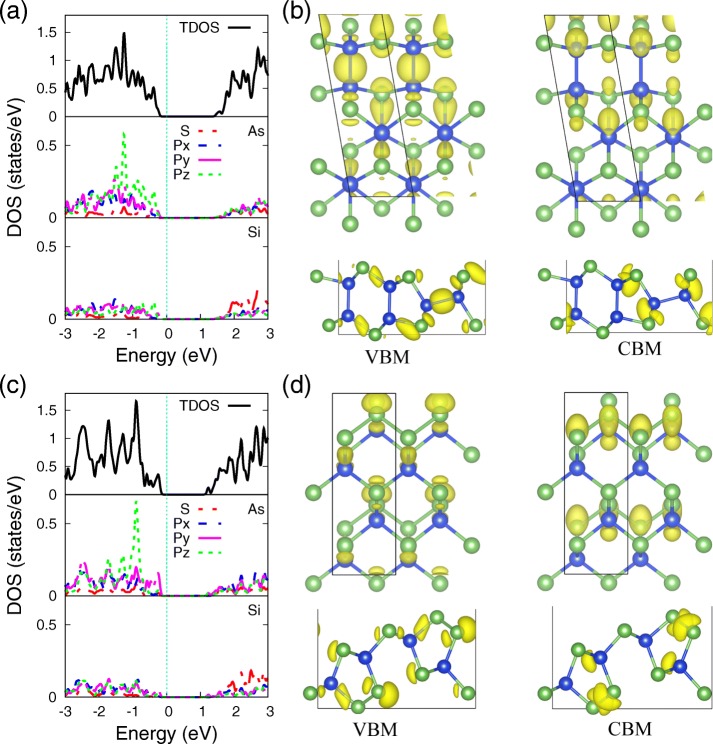
Projected density of states and VBM and CBM’s electron density. The projected density of states (PDOS) of As and Si atoms and the electron density distribution corresponding to VBM and CBM of (**a**, **b**) SiAs and (**c**, **d**) SiAs_2_ monolayers. The isosurface value 0.034 *e*/Å^3^

To further shed light on the underlying bonding mechanism of Si and As atoms in monolayers SiAs and SiAs_2_, the total and partial density of states (PDOS) of them using PBE functional, with their electron density distribution corresponding to VBM and CBM, are provided in Fig. 4, respectively. One can see that the PDOS of As and Si atoms (Fig. 4a, c) shows strong hybridization of *s* and *p* orbitals, indicating the strong covalent bond between them. The distinctions between monolayers SiAs and SiAs_2_ are the localization of *p*_*z*_ orbital, which are attribute to the different bonding coordination environment of As atom. The lone pair electron states, localized at As atom in both of SiAs and SiAs_2_ monolayers, augment the three nearest bonding orbitals to decide the monolayer structure buckling formation and to form the *p*_*z*_ orbital localizing action. In monolayer SiAs, the lone pairs are apart by Si–As bond, which relax the repulsive effect and broaden the *p*_*z*_ orbital. Whereas in monolayer SiAs_2_, As–As bond, remaining the situation which is very common in group V semiconductors, localizes the *p*_*z*_ orbital in a deeper energy level.

As we know, the character of frontier states is not only of interest in a microscopic understanding of the conduction channels but also of great concern for the design of optimal contacts.[52] The charge densities corresponding to VBM and CBM of monolayers SiAs and SiAs_2_ are presented in Fig. 4b and d, respectively. The VBM is almost the hybridization of 3p orbitals of Si and As, while CBM is mainly from the contribution of 3s orbitals of Si and As, which are also consistent with PDOS results in Fig. 4a, c and the electronic orbital decomposition of band structures in Fig. 3.

**Fig. 5:**
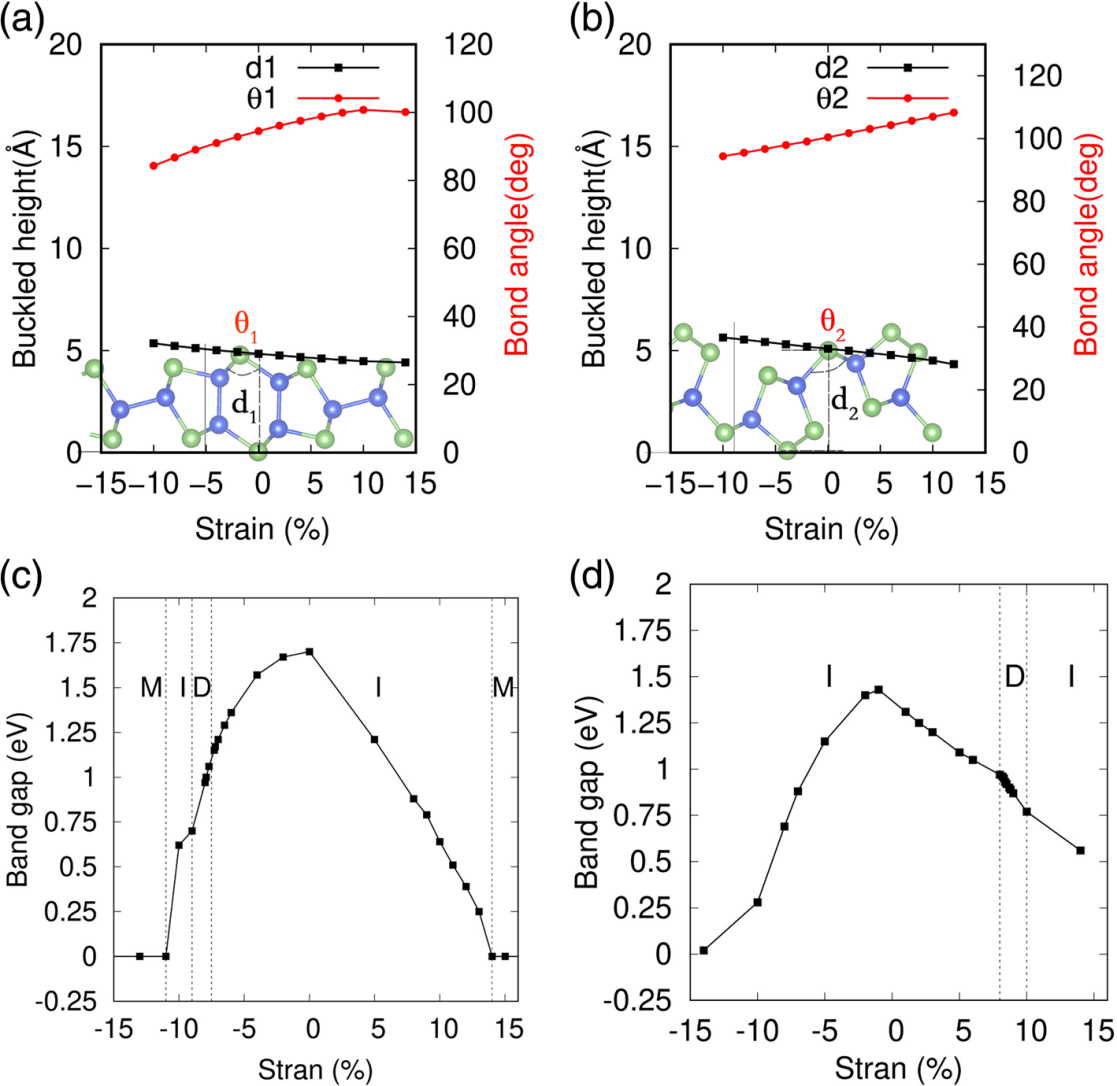
Strain effects on the geometric structures and band gaps of 2D SiAs and SiAs_2_. **a**, **c** represent SiAs; and **b**, **d** denote SiAs_2_; M, I, and D represent metal, indirect semiconductor, and direct semiconductor, respectively

Mechanical strain is an effective way to modulate the electronic properties of 2D materials, which are extensively used to modify the band structure of black and blue phosphorenes and other nanosheet materials [53–55]. Especially, for the buckled structure system, the energy cost is usually quite small to induce a marked strain. Here, the application of mechanical strain is simulated by varying the lattice constant as well as the internal degrees of freedom of each atom during the geometric optimization. The strain *ε* is defined as *ε*=(*l*−*l*_0_)/*l*_0_, where *l* and *l*_0_ are the strained and equilibrium lattice constants of monolayers SiAs and SiAs_2_. In Fig. 5a, b, the detailed variations of high buckling geometric structure of 2D SiAs and SiAs_2_ under strains are represented, respectively. One can see that their buckled heights are expanded or compressed by changed the buckled angle *θ*_1(2)_ with biaxial compressive or tensile strains in nearly linear variations. And we also found that their high buckling geometric structure are both still kept well under quite large strains, whose phonon spectra, as shown in Additional file 1: Figure S5 and S6, exists no negative frequencies even at the large strain regime. The gap variations of monolayer SiAs and SiAs_2_ under biaxial compressive and tensile strains are shown in Fig. 5c, d, respectively. One can see that the electronic properties of SiAs and SiAs_2_ sensitively depend on the strain and undergo an indirect to direct band transition in certain strain region and then to metal at a large strain region.

**Fig. 6:**
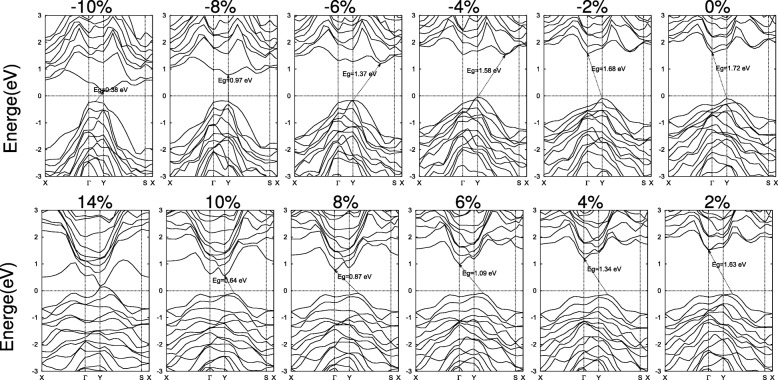
Band structures of 2D SiAs under the biaxial strains. The Fermi level is set as zero and indicated with a dot line

**Fig. 7:**
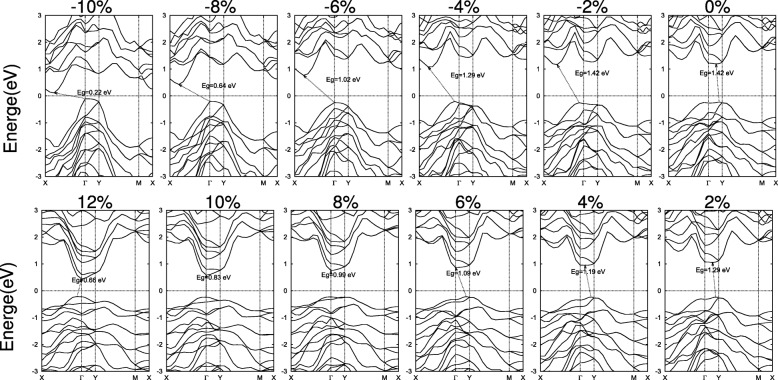
Band structures of 2D SiAs_2_ under the biaxial strains. The Fermi level is set as zero and indicated with a dot line

The detailed variations of SiAs and SiAs_2_ band structures are exhibited in Figs. 6 and 7, respectively. Under biaxial compressive strains, the buckled height of monolayer SiAs is increasing and the CBM shifts from *Γ* to a point on the Y–S line and back to Y. While the VBM is kept still at Y point until the compressive strain reachs *ε* =− 10*%*. Therefore, with increasing compressive strain the band gap switches from indirect Y to *Γ*, via indirect Y to a point on the Y–S line, to direct Y to Y and back to indirect a point on the *Γ*–Y line to Y, as shown in Fig. 6. For tensile strains, the VBM at Y moves to a point on the Y–S line and the CBM at *Γ* moves to Y and the band gap remains indirect. For large strain, no matter compressive or tensile leads to a metal transition, as shown in the Fig. 5c.

In Fig. 7, a similar study has been carried out for 2D SiAs_2_. Instead of compression, tensile strains in the range of 8–10% result in direct band gaps. when the monolayer SiAs_2_ spreads with a decreasing of buckled height under the tensile strains, the VBM shift from a point on the *Γ*–Y line to *Γ* and keep still in the range of 8–10% and then shift apart to a point on the *Γ*–X line, while the CBM moves from a point on the *Γ*–Y line to *Γ* and hold on. Therefore, with rising tensile strain, the band gap switches from indirect on the *Γ*–Y line to direct *Γ*– *Γ* and then back to indirect a point on the *Γ*–X line to *Γ*, as illustrated in Fig. 7. Compressive strains remain the indirect band gap. And large strains have similar effects, leading to a metal transition as SiAs.

Representative direct band structures of strained SiAs and SiAs_2_ are also shown in Additional file 1: Figure S7a and b by the PBE and HSE calculations. For SiAs, a direct band gap of *E*_*g*_ = 1.75 *eV*(HSE) with the VBM and CBM localized at the *Y* points is obtained under a biaxial compressive strain of *ε* = − 7.5*%*. Unlike SiAs, a biaxial tensile strains of *ε*=8.5*%* induces the SiAs_2_ to a direct band of *E*_*g*_ = 1.60 *eV*(HSE). And the VBM and CBM are at the *Γ* point.

## Conclusions

In summary, performing a first-principles DFT calculation, we have propsed two new kinds of 2D materials of silicon and arsenic compound, SiAs and SiAs_2_, which are both dynamically and thermodynamically stable. Our caculations show that SiAs and SiAs_2_ monolayers are indirect semiconductors with the band gaps of 2.39 *eV* and 2.07 *eV*, respectively. The band gap of SiAs and SiAs_2_ monolayers are sensitive to strain, which undergo an indirect to direct band transition and even to metal upon certain mechanical strain. SiAs and SiAs_2_ monolayers possess higher mobility than MoS_2_ and display anisotropic transportation like the black phosphorene. Our works pave a new route at nanoscale for novel functionalities of optical devies.

## Additional file

Additional file 1 Supplementary online material for “Strain tunable bandgap and high carrier mobility in SiAs, SiAs_2_ monolayers from first-principles studies”. (PDF 1507 kb)

## Abbreviations

2D: Two-dimensional
CASTEP: Cambridge sequential total energy package
CBM: Conduction band minimum
DFT: Density functional theory
DFPT: Density functional perturbation theory
DP: Deformation potential
GGA: Generalized gradient approximation
MD: Molecular dynamics
NVT: Moles-volume-temperature
PAW: Projector augmented wave
PBE: Perdew-Burke-Ernzerhof
PDOS: Partial density of states
TMDs: Transiton-metal dichalcogenides
VASP: Vienna ab initio simulation package
VBM: Valence band maximum
