# Supplementary material for: Strain Tunable Bandgap and High Carrier Mobility in SiAs and SiAs2 Monolayers from First-Principles Studies
Source: Nanoscale Res Lett. 2018 Dec 12;13:404. doi: 10.1186/s11671-018-2809-6 (PMC6291413; doi:10.1186/s11671-018-2809-6)
Supplement: Supplementary file 1 — Supplementary online material for “Strain tunable bandgap and high carrier mobility in SiAs, SiAs2 monolayers from first-principles studies”. (PDF 1507 kb) [file 11671_2018_2809_MOESM1_ESM.pdf]

Supplementary Online Material for "Strain tunable bandgap and high carrier mobility in SiAs,$SiAs_2$ monolayers from first-Principles Studies "

Shouyan Bai,[1] Chun-Yao Niu,[1,*] Weiyang Yu,[2] Zhili Zhu,[1] Xiaolin Cai,[2] and Yu Jia[1,3,†]

[1] *International Laboratory for Quantum Functional Materials of Henan, Zhengzhou University, Zhengzhou, 450001, China*

[2] *School of Physics and Electronic Information Engineering, Henan Polytechnic University, Jiaozuo, 454000, China*

[3] *Key Laboratory for Special Functional Materials of Ministry of Education, Henan University, Kaifeng, 475001, China*

(Dated: November 6, 2018)

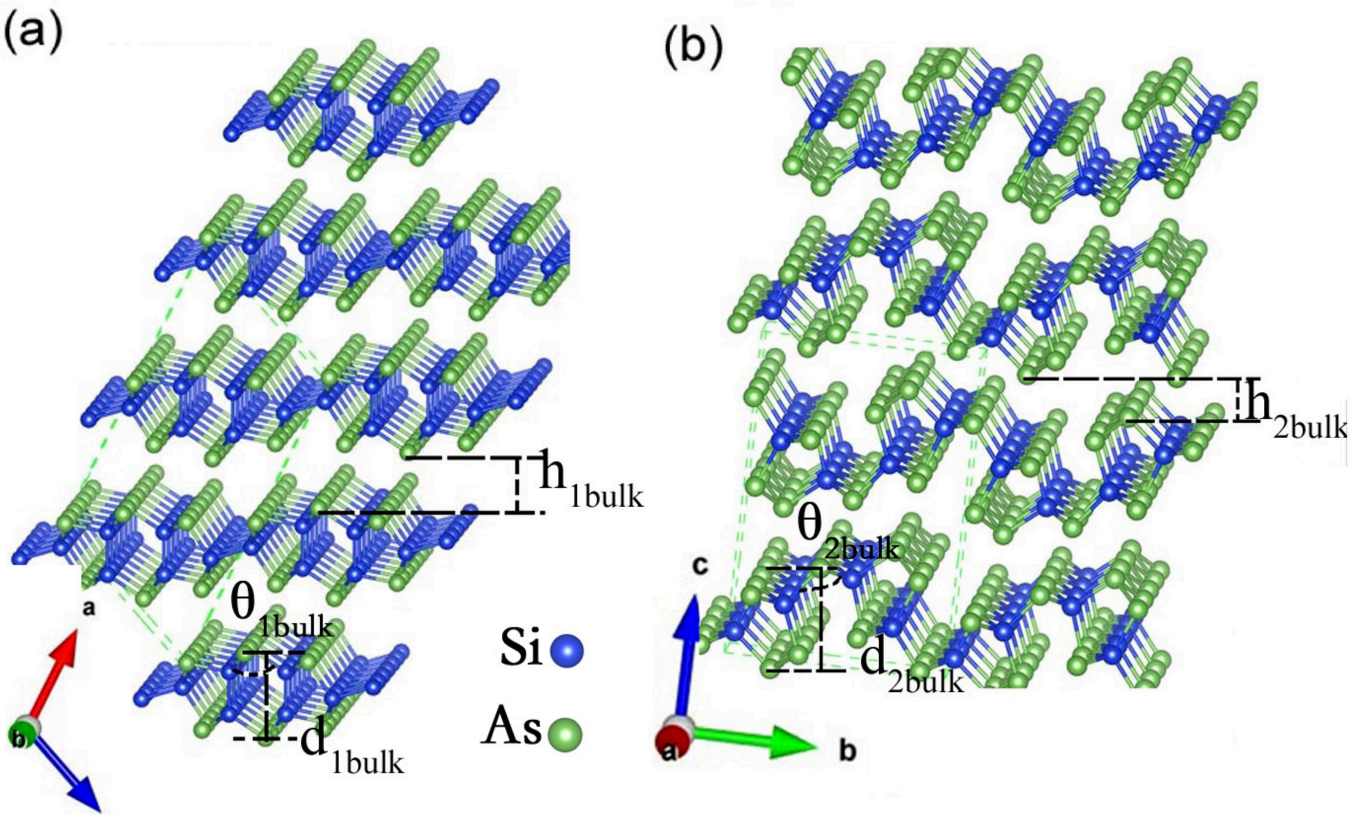

Fig. S 1. Geometrical structures of bulk (a) SiAs and (b) $SiAs_2$, respectively.

TABLE I: The results for optimized geometries of SiAs and $SiAs_2$ bulk and relaxed free-standing monolayers obtained by DFT with PBE exchang-correlation function.

	SiAs	$SiAs_2$
bulk	$d_{1bulk} = 4.79$Å	$d_{2bulk} = 5.03$Å
	$\theta_{1bulk} = 95.26°$	$\theta_{2bulk} = 101.03°$
	$h_{bulk} = 3.06$Å	$h_{2bulk} = 1.66$Å
monolayer	$d_1 = 4.86$Å	$d_2 = 5.09$Å
	$\theta_1 = 94.53°$	$\theta_2 = 100.42°$

Typeset by REVTEX

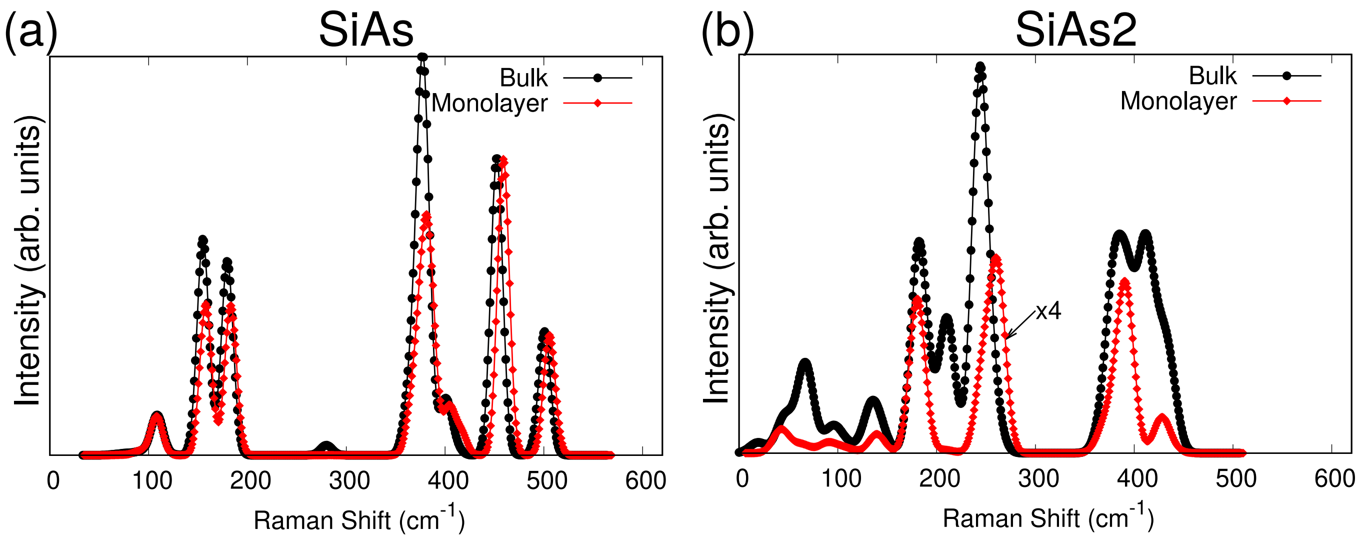

Fig. S 2. Calculated Raman spectra of bulk and monolayer (a) SiAs and (b) $SiAs_2$,respectively.

Accoding to the DP theory, the carrier mobility (μ) of 2D structure can given by:

$$\mu_{2D} = \frac{e\hbar^3 C_{2D}}{k_B T m^* m_d (E_1^i)^2} \tag{1}$$

where k_B is Boltzmann constant, and T is the temperature (300K). m^* is the effective mass in the transport direction, and $m_d = \sqrt{m_x{}^* m_y{}^*}$ is the average effective mass. The term ${E_1}^i$ represents the deformation potential constant of the VBM for hole or the CBM for electron along the transport direction, defined by ${E_1}^i = \Delta V_i/(\Delta l/l_0)$. Here, l_0 is the lattice constant in the transport direction, Δl is the deformation of l_0, and ΔV_i denotes the energy change of VBM or CBM when monolayer SiAs and $SiAs_2$ are under uniaxial strain. The elastic modulus C_{2D} in the x and y directions is derived from $(E - E_0)/S_0 = C(\Delta l/l_0)^2/2$, where E is the total energy and S_0 is the equilibrium area of a 2D SiAs and $SiAs_2$, respectively. We used $\Delta l/l_0$ ranging from -1.0% to 1.0% to fit the values of C_{2D} and E_1^i(Fig. S2 and S3).

* e-mail address:niuchunyao@zzu.edu.cn
† e-mail address:jiayu@zzu.edu.cn

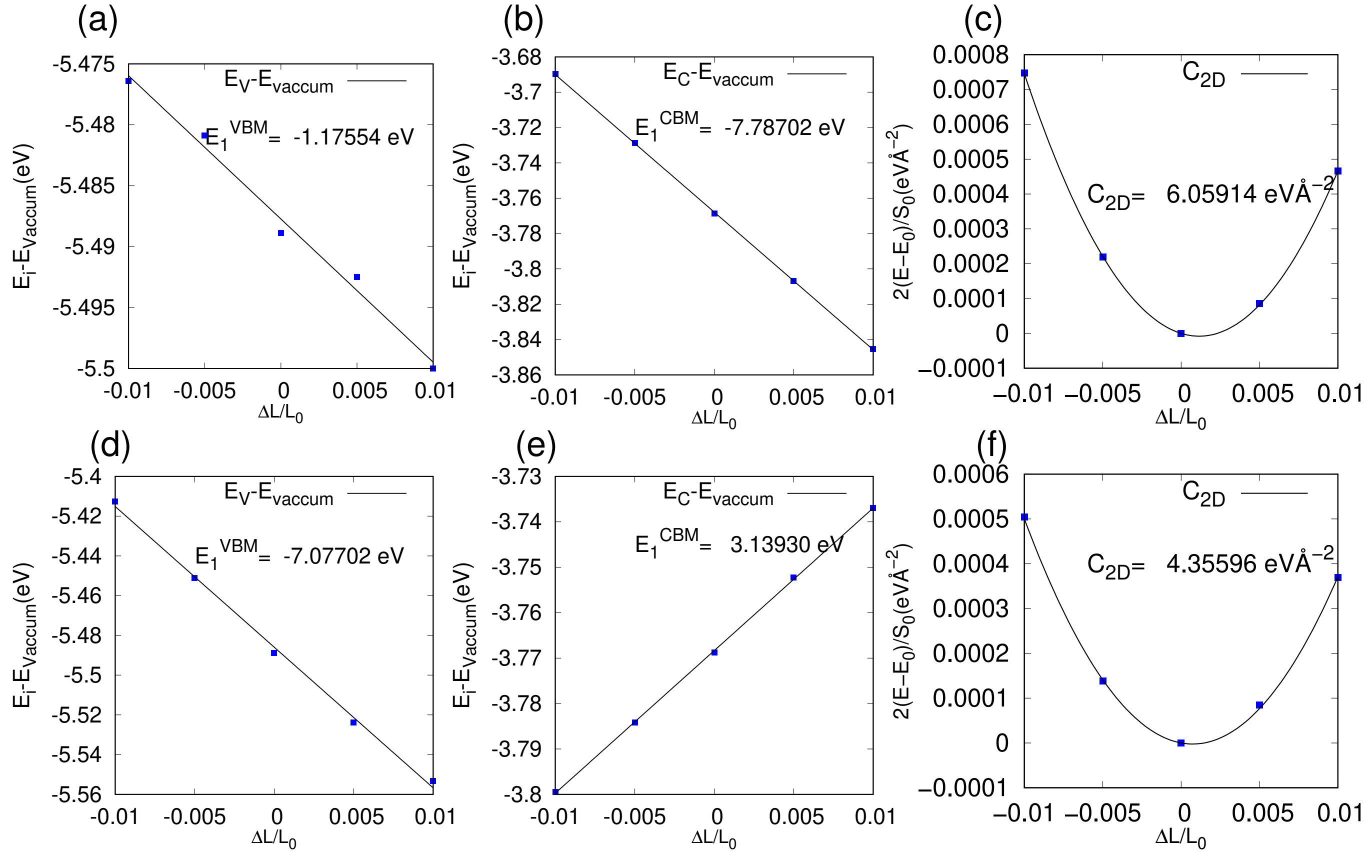

Fig. S 3. Deformation potential constants and elastic modulus of 2D SiAs based on PBE band structures. (a), (b), (c) denote the x direction, and (d), (e), (f) denote the y direction.

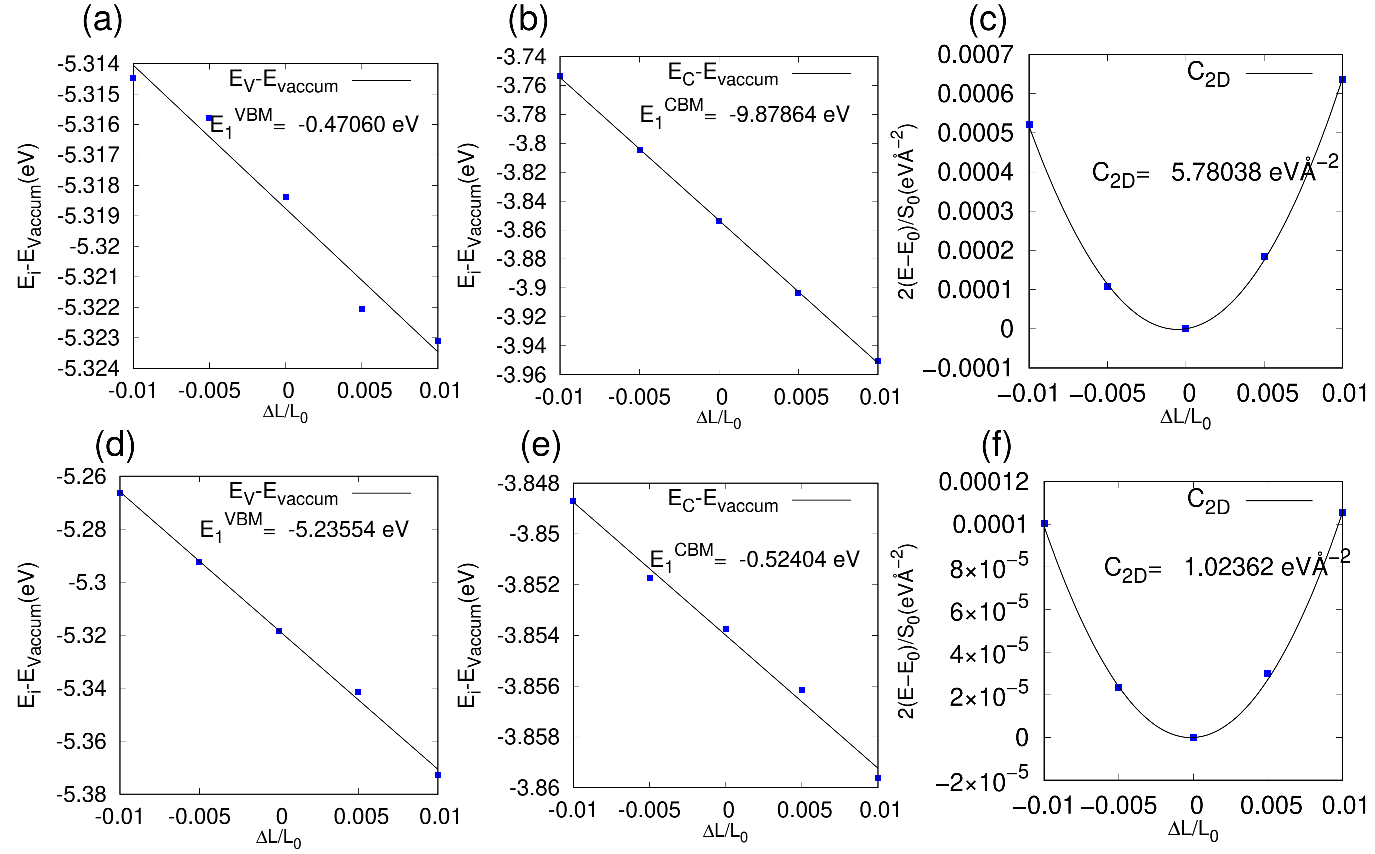

Fig. S 4. Deformation potential constants and elastic modulus of 2D $SiAs_2$ based on PBE band structures. (a), (b), (c) denote the x direction, and (d), (e), (f) denote the y direction.

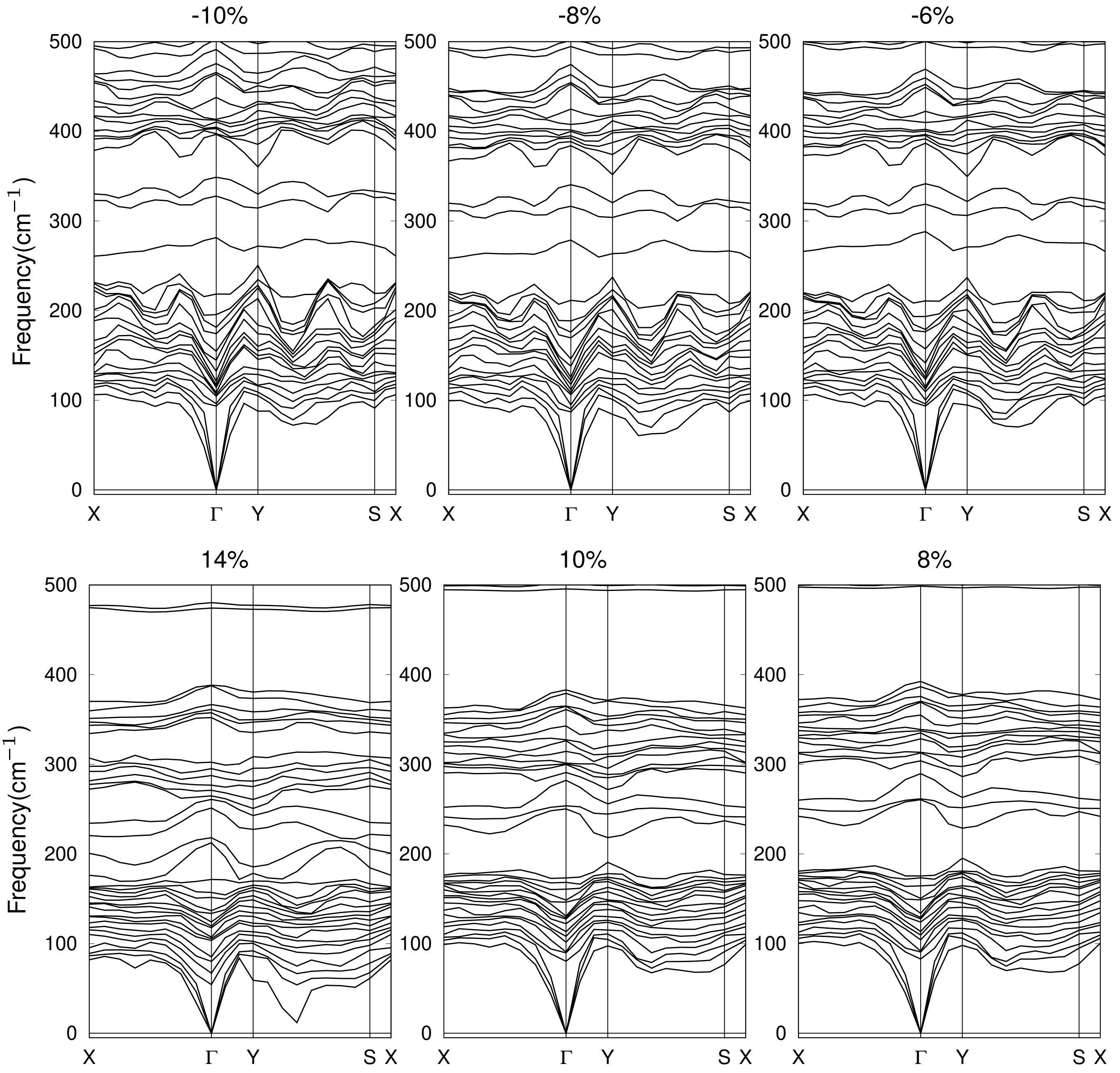

Fig. S 5. Phonon dispersion curves of the proposed 2D SiAs under the biaxial stranins.

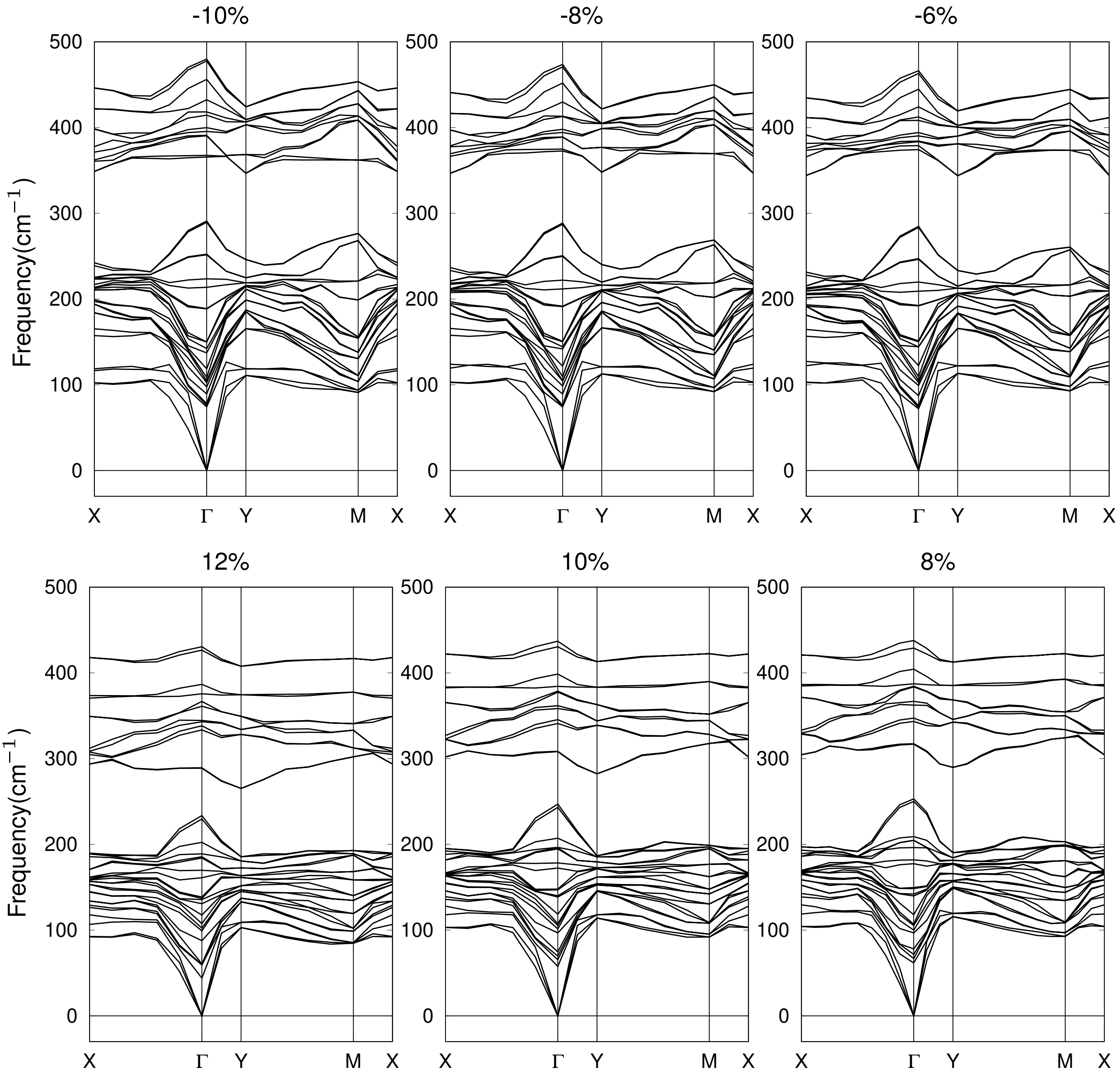

Fig. S 6. Phonon dispersion curves of the proposed 2D $SiAs_2$ under the biaxial stranins.

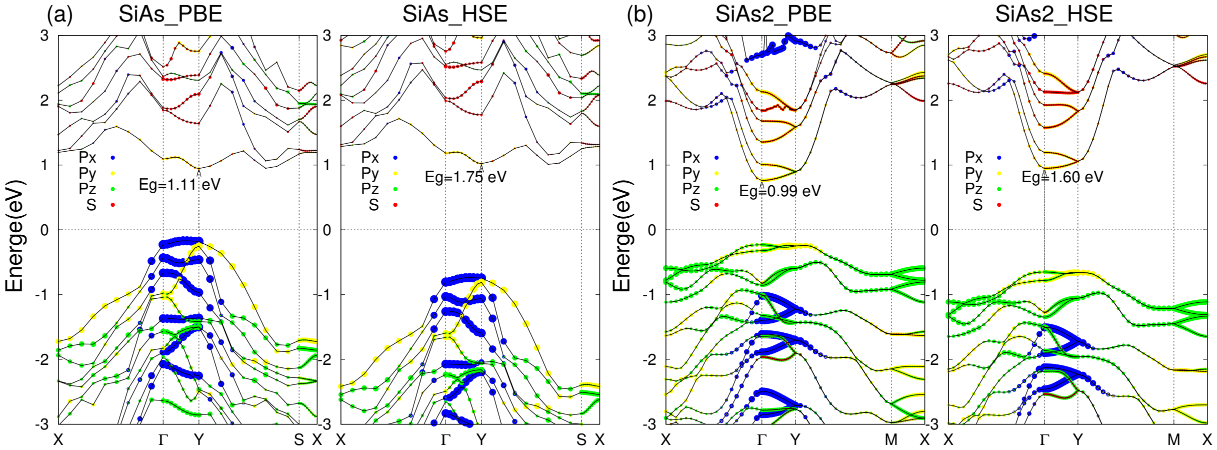

Fig. S 7. (color online). The electronic orbital decomposition of band structures of (a) monolayer SiAs under strain of $\varepsilon = -7.5\%$ and (b) $SiAs_2$ under strain of $\varepsilon = 8.5\%$ calculated by PBE and hybrid functional (HSE06), red is s orbital, while blue, yellow, and green are p_x, p_y, and p_z, respectively. The Fermi level is set as zero and indicated with a dot line. The Fermi level is set to zero, indicated by the horizontal dashed line.
